# In Vivo Ocular Pharmacokinetics and Toxicity of Siponimod in Albino Rabbits

**DOI:** 10.1021/acs.molpharmaceut.4c00063

**Published:** 2024-06-10

**Authors:** Rasha A. Alshaikh, Rania A. Salah El Din, Rania Gamal Eldin Zaki, Christian Waeber, Katie B. Ryan

**Affiliations:** † School of Pharmacy, University College Cork, Cork T12 K8AF, Ireland; ‡ Faculty of Pharmacy, Tanta University, Tanta 31511, Egypt; § Department of Anatomy and Embryology, Faculty of Medicine, Ain Shams University, Cairo 11566, Egypt; ∥ Department of Anatomy and Embryology, Faculty of Medicine, Newgiza University, Giza 12585, Egypt; ⊥ Department of Ophthalmology, Faculty of Medicine, Ain Shams University, Cairo 11566, Egypt; # Department of Pharmacology and Therapeutics, School of Medicine, University College Cork, Cork T12 K8AF, Ireland; ¶ SSPC The SFI Research Centre for Pharmaceuticals, School of Pharmacy, University College Cork, Cork T12 K8AF, Ireland

**Keywords:** diabetes mellitus, age-related macular degeneration, intravitreal administration, neovascularization, siponimod degradation, siponimod stability, ocular half-life

## Abstract

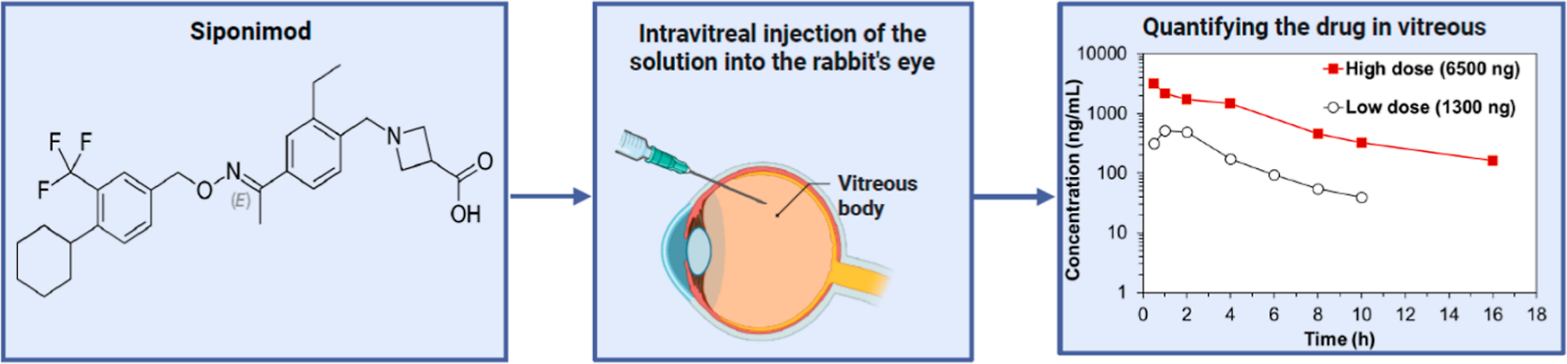

Siponimod is a promising agent for the inhibition of ocular neovascularization in diabetic retinopathy and age-related macular degeneration. Siponimod’s development for ophthalmological application is hindered by the limited information available on the drug’s solubility, stability, ocular pharmacokinetics (PK), and toxicity in vivo. In this study, we investigated the aqueous stability of siponimod under stress conditions (up to 60 °C) and its degradation behavior in solution. Additionally, siponimod’s ocular PK and toxicity were investigated using intravitreal injection of two different doses (either 1300 or 6500 ng) in an albino rabbit model. Siponimod concentration was quantified in the extracted vitreous, and the PK parameters were calculated. The drug half-life after administration of the low and high doses was 2.8 and 3.9 h, respectively. The data obtained in vivo was used to test the ability of published in silico models to predict siponimod’s PK accurately. Two models that correlated siponimod’s molecular descriptors with its elimination from the vitreous closely predicted the half-life. Furthermore, 24 h and 7 days after intravitreal injections, the retinas showed no signs of toxicity. This study provides important information necessary for the formulation and development of siponimod for ophthalmologic applications. The short half-life of siponimod necessitates the development of a sustained drug delivery system to maintain therapeutic concentrations over an extended period, while the lack of short-term ocular toxicity observed in the retinas of siponimod-treated rabbits supports possible clinical use.

## 1 Introduction

Diabetic retinopathy (DR) and age-related macular degeneration (AMD) are among the leading causes of preventable blindness.^1^ Both are characterized by strong angiogenic and inflammatory components^2^ and the release of hypoxia-induced growth factors including vascular endothelial growth factor (VEGF), angiopoietin-2, and sphingosine 1-phosphate (S1P). These growth factors are upregulated in the eyes of AMD and DR patients^3−7^ and trigger the formation of new hyperpermeable blood vessels with defective smooth muscle coverage.^8^

Current pharmacotherapy includes intravitreal injection of angiogenesis inhibitors to neutralize VEGF signaling and extended-release corticosteroids to suppress the inflammatory component that can further contribute to disease progression in DR.^9,10^ Unfortunately, currently used anti-VEGF treatments are associated with many drawbacks, including unsatisfactory gains in visual acuity, instability, high cost, the need for repeated invasive intravitreal injections, and a high rate of treatment resistance (up to 40%).^9,11^ Therefore, there is an unmet clinical need to identify alternative pharmacological targets to inhibit ocular angiogenesis and design sustained-release treatment options that reduce the frequency of intravitreal injections.

Siponimod (BAF-312) is a modulator for S1P receptor 1 (S1PR_1_) and S1P receptor 5 (S1PR_5_). It is approved for managing multiple sclerosis (Mayzent, siponimod fumarate tablets).^12^ S1PR_1_ is highly expressed by endothelial cells and plays a critical role in their proliferation, survival, barrier function, and angiogenic response.^2,13,14^ We have recently shown that siponimod inhibits retinal endothelial cell migration toward serum, VEGF, and S1P, which is a crucial step in blood vessel growth.^15^ Siponimod also attenuates retinal endothelial barrier breakdown and dysfunction associated with leaky endothelium in AMD and DR.^15^ Finally, siponimod inhibits neovascularization and reduces epithelial thinning in a rabbit model of suture-induced corneal neovascularization.^15^ Based on our findings and considering recent reports indicating the ability of siponimod to protect against retinal thinning in multiple sclerosis patients,^16^ we hypothesize that this drug may be of potential benefit in ocular neovascular diseases like AMD and DR. For this use, siponimod would preferentially be administered by intravitreal injection, which is the standard administration route for currently approved treatments and treatments in the pipeline.^10^ Nevertheless, the ocular toxicity, pharmacokinetics (PK) profile, solubility in the vitreous, and thermal stability of siponimod are not known. The lack of this information will hinder further preclinical assessments and clinical trials. Furthermore, PK studies are an integral component of drug development to guide the safe and effective use of new pharmaceuticals in humans, providing critical information on drug dosing, clearance behavior, safety assessment, and aiding in developing suitable drug formulations.^17^

Therefore, we conducted a PK and ocular toxicity study of siponimod in albino rabbits, which are widely used in ophthalmic research due to their similarities to human physiology and anatomy.^18^ Siponimod concentration in the vitreous was measured after a single intravitreal injection to determine how fast siponimod is eliminated. The PK parameters of two drug doses were estimated using noncompartmental analysis (NCA). Retinas were examined 24 h and 7 days after a single-dose administration to detect any harmful effects after the drug injection. We used our PK data as a single-point test of the ability of published in silico models to predict the in vivo intravitreal half-life of the drug based on its molecular descriptors. It is important to highlight that although siponimod is approved by the FDA as a fumarate cocrystal, relatively little is known about the properties of siponimod crystals (free drug crystalline form as opposed to fumarate cocrystals). We, therefore, characterized the thermal stability and both the aqueous and vitreous solubilities of this crystalline form, which are crucial prerequisites for developing ocular dosage forms.

## 2 Materials and Methods

### 2.1 Materials

The siponimod crystal (BAF-312, molecular weight 516.6 g/mol) material was a gift from Novartis Pharma AG, Basel, Switzerland. Acetonitrile (HPLC grade), formic acid, Tween-80, and dimethyl sulfoxide (DMSO) were purchased from Sigma-Aldrich, St. Louis, Missouri or Wicklow, Ireland. Hematoxylin was purchased from BioVision, Waltham, Massachusetts. Eosin was purchased from Sisco Research Laboratories, Mumbai, India. DPX mounting medium was purchased from LOBA Chemie, Mumbai, India. Phosphate-buffered saline (PBS) was purchased from Sigma-Aldrich, Ireland.

### 2.2 Intravitreal Injection and PK Study in Albino Rabbits

Institutional and international guidelines were followed for the care and use of laboratory animals. The experiment was carried out following the European Directive relating to animal experimentation (2010/63/EU). The protocol for the PK study was approved by the Animal Experimentation Ethics Committee of University College Cork (AEEC 2022-004) and the Faculty of Medicine, AinShams University (Approval number R3002022A). The results of the study are reported according to the ARRIVE 2.0 guidelines.^19^

Good practice recommendations for the conduct of ocular PK studies were followed to help ensure the reliable calculation of PK parameters.^18^ These included (1) the introduction of siponimod in solution, (2) at least six time points were reported for each PK profile, (3) the first data point was recorded at 0.5 h after intravitreal injection, (4) the recorded data points covered at least three half-lives of the drug, and (5) between 3 and 5 replicate eyes were used at each time point.^18^ The experiments were initiated with five rabbits at each time point; this number was reduced to three, as the observed standard deviation was lower than expected.

Adult New Zealand albino rabbits, obtained from a certified private breeder, (50% males and 50% females) weighing approximately 2.5 kg were acclimatized for a minimum of 10 days in the MASRI animal facility at AinShams University. Before intravitreal injection, the rabbits were anesthetized using xylazine hydrochloride (im, 5 mg/kg) and ketamine hydrochloride (iv, 35 mg/kg). The respiratory rate and depth and body temperature were monitored during anesthesia and after recovery from anesthesia. The ocular surface was anesthetized with benoxinate hydrochloride eyedrops (BENOX, 0.4%). Drug solution (100 μL) containing either 1300 or 6500 ng of siponimod was injected into the right rabbit vitreous using a 30 G needle. The drug was dissolved at 40 °C in saline supplemented with 0.08% DMSO and 0.0015% Tween-80. The same volume of vehicle was administered to the left eye as a control.

After injection, the rabbits were allowed to recover from anesthesia at 30 °C. The breathing rate and rectal temperatures were closely monitored until the animals were ambulatory. Rabbits (*n* = 2–5, at each time point) were euthanized with an overdose of ketamine hydrochloride (300 mg/kg) and xylazine hydrochloride (30 mg/kg) 0.5, 1, 2, 4, 6, 8, 10, 16, and 24 h after receiving the intravitreal injection. Rabbits euthanized at 24 h received an additional intravitreal injection of 100 μL of the vehicle into their left eye to examine potential solvent toxicity (control eyes). Rabbits were enucleated at each time point, and samples from the vitreous were collected to determine siponimod concentration.

### 2.3 Dose Selection

Siponimod doses were selected based on our previous work on human retinal endothelial cell lines,^15^ which showed robust antiangiogenic properties with 1 μM siponimod, and no adverse effect on retinal endothelial cell viability or toxicity. Assuming a vitreous volume of 3.5 mL in the relevant (i.e., aging) population,^20−23^ we estimated that 1800 ng of siponimod would yield a 1 μM concentration immediately after injection. As previously suggested, the rabbit dose should be 28.6% lower than the human dose due to different vitreous volume and elimination kinetics in this species.^18,24^ Therefore, we selected the first dose of 1300 ng of siponimod (low dose).^18,24^ We also tested a 5 times higher dose of siponimod (6500 ng) to investigate if the higher dose exhibits a different PK profile or possible toxicity, which aligns with the approach adopted in a previous study evaluating PK and toxicity of different triamcinolone doses.^25^ In this study, testing higher doses of the drug >6500 ng was hindered by the solubility limit of siponimod in aqueous solution. Higher doses of lipophilic molecules may exceed the saturation solubility in the vitreous, leading to spontaneous precipitation which can change the elimination rate of the drug.^25^

### 2.4 Vitreous Sample Collection and Drug Extraction for Quantification

Vitreous samples were mixed with an equal volume of 100% acetonitrile for sample deproteinization. The mixture was left for 10 min before 15 min centrifugation at 14,000 rpm. The precipitation–centrifugation steps were repeated if needed to ensure a clear supernatant was obtained. The supernatant was collected, and the sample volume was reduced under vacuum for a minimum of 90 min. After being dried, the samples were collected, and the volume was adjusted using the mobile phase before HPLC analysis. A control sample of siponimod solution was processed in the same way to confirm that the deproteinization and volume reduction steps did not affect the siponimod stability.

### 2.5 HPLC Analysis

The concentration of siponimod in the vitreous samples was determined by using an HPLC system (1260 infinity chromatographic system, Agilent Technologies, Santa Clara, California) coupled with a UV/vis detector. The stationary phase was a reverse C18 Eclipse Plus column (4.6 mm × 150 mm, average particle size 5 μm) (Agilent technologies). The mobile phase consisted of 45% water acidified with 0.5% v/v formic acid (pH ≈ 3.0 ± 0.1) and 55% acetonitrile with a 1 mL/min flow rate. The column temperature was maintained at room temperature, and the effluent was monitored at 220 nm. Serial dilutions of standard siponimod working solutions with concentration ranges of 0.05–0.5 and 1–20 μg/mL were prepared in the mobile phase and used to develop the respective calibration curves (CC1 and CC2) for each range. Each calibration curve was based on at least five standard concentrations, with each concentration analyzed in duplicate.

For each concentration range, the established HPLC method was validated for intraday and interday accuracy (% recovery), intraday and interday precision (% relative standard deviation), limit of detection (LOD), and limit of quantitation (LOQ). Parameters were calculated using three calibration curves analyzed within the same day and three calibration curves analyzed over three consecutive days (*n* = 6).^26^ Unknown siponimod concentrations were calculated by interpolation from the established calibration curves by using chromatogram peak areas. All unknown samples were analyzed in duplicate.

### 2.6 Assessment of Retinal Toxicity by H&E Staining

Siponimod ocular toxicity was assessed by H&E staining of the retinas 24 h and 7 days after intravitreal injection. At each time point, rabbits (*n* = 3) were euthanized, the eyes were surgically removed and immediately injected with and stored in 10% buffered formalin for a minimum of 48 h before further processing as follows. The eyeballs were halved along the coronal plane, processed, and embedded in paraffin blocks. Sections of 5 μm thickness were mounted onto glass slides for H&E staining to detect gross histopathological changes in retinal layers as described previously.^27^ Sections were dewaxed using two successive rinses in xylene before being rehydrated in an ethanol series (5 min each in 100, 90, 70, and 0% ethanol). The sections were submerged in hematoxylin stain (prepared per manufacturer’s instructions) before being washed in tap water. Next, the sections were counterstained with eosin and dehydrated in an ethanol series (70, 90, and 100% ethanol) each for one min, followed by rinsing in two changes of xylene, for 5 min each. Then, a DPX mounting medium was added before a coverslip was placed on the sections. Images were acquired using an OLYMPUS BX43 microscope (OLYMPUS, Tokyo, Japan). Three random images were acquired for each slide, and the acquired images were then assessed for signs of retinal toxicity. Image acquisition and analysis were conducted by blinded investigators.

### 2.7 Collection of Pig Vitreous and Determination of Siponimod Equilibrium Solubility

The solubility of siponimod was determined in porcine vitreous, using porcine eyes that were collected from euthanized pigs after completion of other ethically and regulatory approved studies at University College Cork. Vitreous humor was collected from the eyes, pooled, and centrifuged at 4000 rpm for 10 min (Rotanta 460r centrifuge, Hettich, Tuttlingen, Germany) to remove cell debris. The supernatant was recovered, immediately frozen, and stored at −80 °C before use. Despite interspecies variations in anatomy and physiology, both rabbits and pigs exhibit comparable ocular characteristics, rendering them suitable models for ocular research.^28,29^ For ethical reasons, the solubility of siponimod was therefore determined in porcine vitreous, using eyes collected from euthanized pigs after completion of other ethically and regulatory approved studies at University College Cork.

To determine siponimod's solubility, the frozen vitreous was allowed to thaw at room temperature before use. Approximately 5 mL of vitreous or PBS was placed in glass vials, and an excess amount of siponimod (10 mg) was added to each vial. The vials were vortexed for 2 min and then stirred at 200 rpm for 96 h at room temperature using a magnetic stirrer (Heidolph MR3001K, Heidolph Instruments GmbH, Schwabach, Germany). At predetermined time points (24, 48, 72, and 96 h), two samples of the solution were taken and immediately centrifuged at 14,000 rpm for 15 min before being filtered using a 0.22 μm cellulose acetate filter to remove any undissolved drug particles. Filtered samples were then processed as described in Section 2.4 before quantifying siponimod using the established HPLC method. Each experiment was performed in triplicate (*n* = 3).

### 2.8 Siponimod Thermal Stability

To assess the thermal stability in an aqueous solution, siponimod was dissolved in PBS (pH 7.4) at a final concentration of 53.9 ± 4.8 μg/mL. Drug solutions were then incubated in airtight containers and stored at refrigeration temperature (4 °C), room temperature (25 °C), 40, or 60 °C (Binder drying chamber, BINDER GmbH, Tuttlingen, Germany). Samples (1 mL) were taken at predetermined time points (0, 4, 7, 21, and 45 days) and analyzed using the HPLC method described above to quantify the drug concentration.

### 2.9 Investigation of the Main Degradation Product(s) of Siponimod in Solution Using LC–MS

LC–MS analysis was conducted using a Waters 2695 Separations Module equipped with a Waters 2996 Photodiode Array Detector, which was connected to a Waters Quattro micro-TM API mass spectrometer (Instrument no. QAA1202, Waters Corporation, Massachusetts, United States) operating in positive ionization mode (ESI+, *m*/*z* = 100–1000). An isocratic mobile phase consisting of acetonitrile (55%) and 0.5% v/v formic acid in water (45% v/v) was employed at a flow rate of 1.0 mL/min, with a total run time of 15 min. A 20 μL volume of freshly prepared siponimod solution or selected degradation sample was injected into an Eclipse Plus C18 HPLC column (4.6 × 150 mm, average particle size 5 μm, Agilent Technologies) at 20 °C. The eluted components were monitored at 220 and 278 nm before being split into two streams flowing at approximately 0.5 mL/min each, with one stream directed into the mass spectrometer electrospray ionization chamber and the other discarded as waste. For MS detection in ESI+ mode, the following settings were used: source temperature, 130 °C; desolvation temperature, 350 °C; nitrogen desolvation gas flow rate, 500 L/h; cone gas flow, 25 L/h; capillary potential, 3.6 kV; cone potential, 18 V. Data were acquired using a scan mode covering the *m*/*z* range of 100–1000 in 1 s intervals. The acquired data were recorded and processed using Masslynx Mass Spectrometry 4.1 software (Waters Corporation, Massachusetts, United States).

### 2.10 Data Analysis

The statistical analysis included all the data obtained, and no data points were excluded. The PK data were analyzed using the *PKSolver 2.0* add-in program for Microsoft Excel as previously described.^30−34^ A standard NCA was employed using mean drug concentration to estimate the area under the curve (AUC_0–∞_) using the linear trapezoidal method, the terminal half-life (*T*_1/2_), the volume of distribution at the steady state (V_d_), and vitreous clearance (Cl_ivt_). NCA allows the estimation of the PK parameters directly from the measured concentrations with fewer assumptions regarding body compartments^35,36^ and has been employed to estimate the ocular PK of many small and large molecules.^37−40^ As only one data point was obtained from each animal, the PK parameters are reported as mean values. The variance and standard deviation AUC_0–∞_ were calculated using previously reported methods for calculating standard deviation for PK studies with destructive measurement techniques.^41−43^

## 3 Results and Discussion

### 3.1 HPLC Method Validation

The HPLC method was tested for linearity, assay range, precision, accuracy, LOD and LOQ. The results of each of these parameters are summarized in Table 1. As per ICH guidelines,^26^ the linearity of the analytical method was indicated by visual inspection of the calibration curves and by the coefficient of determination (*R*^2^) of the straight line fitted to points of the average calibration standard. The developed methods demonstrated high intraday and interday accuracy, as indicated by the recovered drug percentage (% recovery), which ranged from 98.37 to 104.42% and 98.05 to 102.84 for CC_1_ and CC_2_, respectively (Table 1). The established HPLC methods also demonstrated high within- and day-to-day repeatability (as indicated by the relative standard deviation in Table 1).

**Table 1:** Validation Parameters Including Sensitivity, Linearity, Accuracy, and Precision for the HPLC/UV Method Developed for the Detection and Quantification of Siponimod^a^

		CC_1_	CC_2_
concentration range		0.05–0.5 μg/mL	1–20 μg/mL
slope		267.01 ± 9.50	267.25 ± 3.63
intercept		–1.35 ± 0.81	2.74 ± 18.02
LOD (μg/mL)		0.01	0.22
LOQ (μg/mL)		0.03	0.67
accuracy (% recovery)	intraday	98.37–101.42	98.05–102.84
	interday	99.30–104.44	98.66–100.68
precision (% RSD)	intraday	1.8–6.7	1.54–3.85
	interday	1.5–7	1.13–5.68

a LOD: limit of detection, LOQ: limit of quantitation, and % RSD: percentage relative standard deviation. Slope and intercept values are presented as mean ± standard deviation.

### 3.2 Siponimod PK Profile after Intravitreal Injection

**Figure 1:**
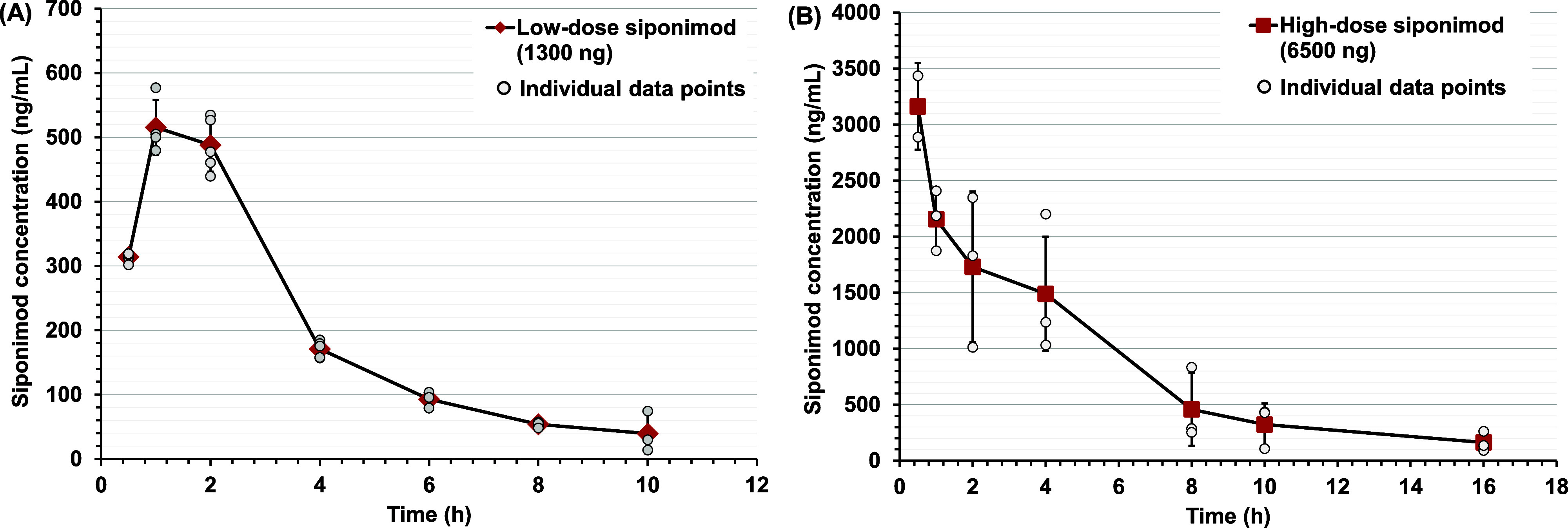
Siponimod concentration (ng/mL) measured in the vitreous of albino rabbits at different time points after intravitreal injection of (A) low-dose (1300 ng) and (B) high-dose siponimod (6500 ng). Data are presented as mean ± standard deviation of the observed values (large red symbols), with small circles showing siponimod concentration obtained from each rabbit, *n* = 2–5 rabbits at each time point.

The intravitreal PK of siponimod were investigated following a single intravitreal injection of siponimod solution containing either 1300 or 6500 ng. The profiles of vitreous concentration over time for the low and high siponimod doses are presented in Figure 1A,B, respectively. The mean intravitreal concentrations of siponimod at different time points are listed in Table 2.

**Table 2:** Mean Siponimod Concentrations in the Vitreous Samples Obtained at Different Time Points for Low and High Doses of the Drug^a^

time (h)	low dose siponimod (1300 ng)		high dose (6500 ng)	
	mean concentration ± (SD) (ng/mL)	number of rabbits (*n*)	mean concentration ± (SD) (ng/mL)	number of rabbits (*n*)
0.5	314.1 (7.4)	5	3161.7 (387.1)	2
1	515.6 (42.6)	4	2156.3 (268.5)	3
2	487.9 (41.5)	5	1730.3 (673.9)	3
4	171.0 (12.8)	5	1489.7 (509.5)	3
6	92.9 (12.6)	3	—	—
8	53.9 (3.5)	5	457.6 (325.6)	3
10	39.4 (31.3)	3	322.5 (187.3)	3
16	nd	3	162.8287 (88.377)	3
24	—	—	nd	3

a Values are presented as mean concentration ± standard deviation (SD), nd: not detected using the established HPLC analysis method, *n* = 2–5 for each time point. The vitreous concentration after injection of the low dose (1300 ng) and high dose of siponimod (6500 ng) was not measured at 24 and 6 h, respectively.

After intravitreal injection of the low dose (1300 ng/mL), the siponimod concentration showed an increase in concentration between 0.5 and 1 h (*T*_max_ at 1 h), followed by a steady decline in the drug concentration. This behavior was not noticed after administration of the high dose (6500 ng), where *C*_max_ was achieved at the first sampling point of 0.5 h (*T*_max_). The profile of the high siponimod dose aligns with anticipated PK behavior for drugs administered intravitreally, as there is no absorption phase. Consequently, the drug concentration promptly decreases through distribution and elimination processes. However, the low drug concentration at *t* = 0.5 h after the injection of the low siponimod dose can be attributed to sampling or analysis error or other processes that might limit free drug concentration at the first time point (e.g., drug precipitation, diffusion, protein binding, or other unknown processes).

**Figure 2:**
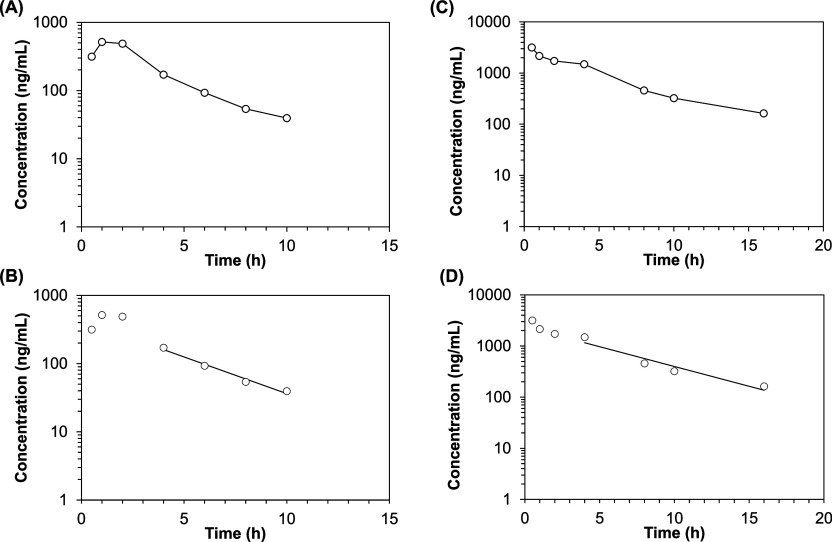
Semilogarithmic plot of concentration–time profiles of siponimod in the vitreous after intravitreal injection of low-dose (1300 ng) (A,B) and high-dose siponimod (6500 ng) (C,D). B,D show the terminal elimination phase. Data are presented as the mean concentration at each time point, and graphs were generated using the PKSolver 2.0 add-in program for Microsoft Excel.

The estimated PK parameters of low and high doses of siponimod by using NCA are presented in Table 3. The Log siponimod concentration vs time profile and the terminal elimination phase are presented in Figure 2. No siponimod was detected in the vitreous of the low-dose or high-dose groups at 16 and 24 h, respectively. No siponimod was detected in the contralateral vitreous (non-injected eye) at sampling times up to 4 h. Following the administration of 1300 ng of siponimod, the half-life (*T*_1/2_) and clearance (CL) were estimated as 2.80 h and 0.59 mL/h, respectively. The *T*_1/2_ and CL for the high siponimod dose (6500 ng) were 3.88 h and 0.42 mL/h, respectively (Table 3). Both high and low doses of siponimod showed a short half-life and comparable clearance from the vitreous.

**Table 3:** PK Parameters of Siponimod in Albino Rabbits, Estimated Using NCA^a^

parameter	low dose	high dose
dose (ng)	1300	6500
*R*	–0.99	–0.96
*C*_max_ (ng/mL)	515.56 ± 42.57	3161.68 ± 387.11
*T*_max_ (h)	1	0.5
*T*_1/2_ (h)	2.80	3.88
*V*_d_ (mL)	2.15	2.08
AUC_0–∞_ (ng/mL h)	2188.29 ± 49.51	15485.20 ± 1444.10
CL (mL/h)	0.59	0.42

a *C*_max_ is the maximum siponimod concentration in the vitreous after intravitreal injection. *T*_max_ is the time of *C*_max_, *T*_1/2_ is the elimination half-life in hours (h), *V*_d_ is the observed volume of distribution at the steady state, AUC_0–∞_ is the area under the curve, and CL is the observed clearance from the vitreous. *C*_max_ is presented as mean ± standard deviation. AUC_0–∞_ is presented as mean value ± standard deviation estimated using previously reported methods for calculating standard deviation for PK studies with destructive measurement techniques.^41−43^

Intravitreal injection introduces the drug into a clear gel-like matrix composed mainly of water with traces of collagenous and non-collagenous proteins.^10,44^ The elimination of the drugs from the vitreous depends on drug diffusion from the injection site through the vitreous gel to the elimination sites, which are the posterior elimination route (blood-ocular barriers) and the anterior elimination route (aqueous humor flow).^44^ The short half-life of siponimod after intravitreal injection is expected, considering its small molecular weight (516.6 g/mol). Small, lipophilic molecules are readily cleared from the vitreous as they can diffuse freely through the vitreous matrix into the paracellular spaces of the inner and outer blood-ocular barrier, where they get eliminated by the systemic circulation. In comparison, the blood-ocular barrier represents a strong barrier for macromolecules (>2 nm) that are mainly eliminated via the aqueous humor flow, resulting in a longer residence time in the eye.^44,45^ Siponimod’s elimination suggests that after administration of the higher (6500 ng) dose of siponimod, the drug concentration in the vitreous will reach its EC_50_ value of 0.2 ng/mL at 52.5 h after injection (where EC_50_ is the drug concentration producing 50% of the maximal response at S1PR_1_).^46^ Therefore, to maintain therapeutic efficacy, a drug solution would need to be administered nearly every 48 h, which would be clinically unacceptable. This necessitates the development of a sustained delivery system if the drug is to be used for ocular applications.

It is important to highlight that the intravitreal half-life of siponimod we obtained in vivo significantly differs from its reported systemic half-life (*T*_1/2,systemic_) after oral administration (approximately 30 h) in humans.^47^ Such a difference is to be expected since different administration routes introduce the drug to sites with different compositions, volumes, distribution mechanisms, metabolic processes, and elimination mechanisms.

### 3.3 Prediction of the Siponimod Half-Life Based on Molecular Descriptors Using Established Mathematical Models

PK experiments in vivo remain the most accurate method to determine the ocular PK parameters of drugs under investigation.^18^ These experiments require a large number of animals as sampling from the vitreous is a terminal process. Therefore, various attempts have been made to build mathematical models to predict the ocular PK of a molecule based on its molecular characteristics. In the following section, molecular descriptors of siponimod (Table 4) were used to predict its vitreous half-life or clearance using different in silico models (Table 5). The calculated values were compared to the practical values obtained in vivo to investigate how well these descriptors can predict the drug elimination kinetics from the vitreous.

**Table 4:** Molecular Descriptors of Siponimod

descriptor	value	source
PubChem CID	44,599,207	PubChem
molecular weight	516.6 g/mol	PubChem
molecular formula	C29H35F3N2O3	PubChem
hydrogen bond donor count (Lipinski)	1	ChEMBL
hydrogen bond acceptor count (Lipinski)^a^	5	ChEMBL
rotatable bonds count	9	ChEMBL
Log *P*	4.76	predicted by XLogP3 3.0
Log *P*	5.85	predicted by ALOGPS 2.1
Log *D* at pH 7	4.3	predicted by Chemaxon logD predictor
Log *D* at pH 7.4	4.28	ChEMBL
topological polar surface area	62.1 Å^2^	obtained from PubChem and computed by Cactvs 3.4.8.18
mass intrinsic solubility	1.2 × 10^–^^4^ g/L at pH 7 and 25°C	obtained from SciFinder scholar. Values are calculated by Advanced Chemistry Development (ACD/Laboratories) Software V11.02 (© 1994–2023 ACD/Laboratories)

a Different counts for hydrogen bond acceptors are present in different databases as the weak hydrogen bond accepting abilities of fluorine atoms are sometimes taken into account. The five hydrogen bond acceptor count denotes all oxygen and nitrogen atoms in the molecule as previously described.^53^

**Table 5:** Different Mathematical Models to Predict the Vitreous Half-Life (*T*_1/2_) or Clearance (CL_ivt_) and Their Application Using Siponimod

no.	equation	molecular descriptors in the equation	calculated *T*_1/2_ or CL_ivt_ for siponimod^a^	references
1	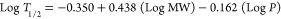 1	MW: molecular weight, *P*: partition coefficient, and *D*: distribution coefficient	0.78 h	(48)
2	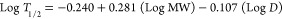 2		1.16 h	
3	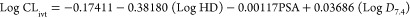 3	HD: hydrogen bond donor count, PSA: polar surface area, and Log *D*_7.4_: distribution coefficient measured at 7.4	0.75 mL/h	(49)
4	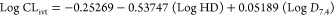 4		0.52 mL/h	
5	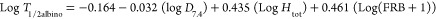 5	Log *D*_7.4_: distribution coefficient measured at 7.4, *H*_tot_: total hydrogen bond count, and FRB: freely rotating bond count	3.14 h	(50)

a *T*_1/2_ was calculated in hours (h) using eqs 1, 2 and 5 and CL_ivt_ was calculated in mL/h using eqs 3 and 4. Siponimod molecular descriptors are summarized in Table 4.

The first two equations were derived by Durairaj et al. to predict the *T*_1/2_ of intravitreally injected drugs in rabbits (eqs 1 and 2, Table 5).^48^ Using both models, siponimod’s calculated half-lives were 0.78 and 1.16 h for the Log *P* (eq 1)- and Log *D* (eq 2)-based models, respectively. These values are considerably lower than the *T*_1/2_ of siponimod that we measured in vivo (2.80–3.88 h, Table 3). These equations do not consider different factors (e.g., molecular flexibility and molecular interaction) that can affect the diffusion of the molecule through the vitreous to the posterior elimination routes.

Del Amo et al. derived two equations to predict the drug clearance from the vitreous based on a data set of small molecular weight compounds (<1449 Da) (eqs 3 and 4, Table 5).^49^ The calculated clearance of siponimod was 0.75 and 0.52 mL/h for eq 3 and eq 4, respectively. These values are close to the in vivo CL_ivt_ of siponimod obtained in our experiments (0.59 and 0.42 mL/h for low and high doses of siponimod, respectively). Both equations assume a positive correlation between the clearance and lipophilicity, which highlights that the drug’s higher solubility in biomembranes (e.g., blood-retinal barrier) contributes to increased intravitreal clearance. The equations also assume a negative correlation between the clearance and the hydrogen bond donor count and polar surface area. Hydrogen bonding between the molecule’s surface and the negatively charged proteins in the vitreous or cell membrane can hinder molecular diffusion and clearance.^49^

Finally, Kidron et al. derived an equation to predict the vitreous *T*_1/2_ of molecules with a molecular weight below 1449 Da (eq 5, Table 5).^50^ The calculated *T*_1/2_ for siponimod using this model was 3.14 h, comparable to the in vivo *T*_1/2_ values of 2.80 and 3.88 h for low and high doses of siponimod, respectively.

By applying the different models, good agreement was found between the calculated values using equations that integrate lipophilicity and hydrogen bond formation ability, with or without molecular flexibility, to predict the drug clearance from the vitreous.^49,50^

### 3.4 Histopathological Examination of H&E-Stained Retinal Sections

**Figure 3:**
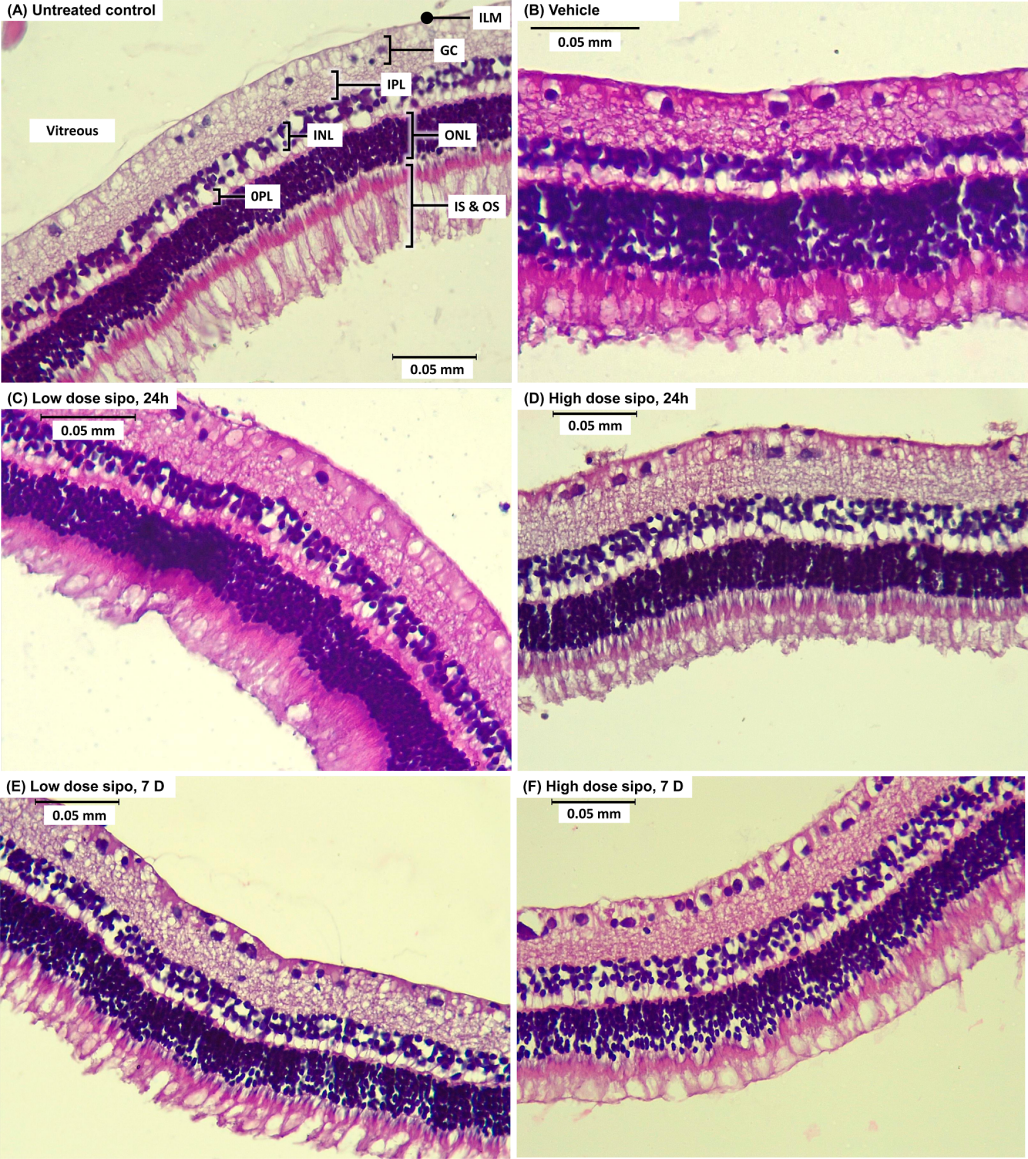
Representative retinal sections were stained with H&E 24 h (A–D) and 7 days (E,F) after intravitreal injection. (A) Untreated control, (B) vehicle control, (C) low dose siponimod (1300 ng) after 24 h, (D) high dose siponimod (6500 ng) after 24 h, (E) low dose siponimod (1300 ng) after 7 days, and (F) high dose siponimod (6500 ng) after 7 days. The sections show no noticeable difference in retinal morphology between groups. ILM, inner limiting membrane; GC, ganglionic cell layer; IPL, inner plexiform layer; INL, inner nuclear layer; OPL, outer plexiform layer; ONL, outer nuclear layer; IS, inner segment of photoreceptors; OS, outer segments of photoreceptors. Scale bar = 0.05 mm.

No ocular or systemic adverse effects were observed in the treated rabbits during the study. Retinal sections of rabbits administered with the low siponimod dose (1300 ng), high siponimod dose (6500 ng), and vehicle were examined for signs of toxicity 24 h and 7 days after the intravitreal injection (Figure 3). The untreated control displayed healthy retinal architecture (Figure 3A), with well-ordered retinal layers.^51^ The retinas of rabbits administered with 100 μL of the vehicle were similar to those of the untreated control group, with no signs of toxicity 24 h after administration (Figure 3B). Siponimod (low and high doses) did not cause any detectable changes in the retinal architecture after 24 h, with sections showing a normal retinal appearance, well-ordered layers, and no signs of vacuoles, inflammation, or edema (Figure 3C,D). To examine any potential delayed toxicity, rabbits were left for 7 days after intravitreal administration of the vehicle or siponimod before being euthanized. Representative images showing the H&E staining of these retinas are presented in Figure 3E,F. Neither dose of siponimod caused any noticeable changes in the retinal architecture. The retinas of the treated animals were comparable to those of the control, showing well-organized layers and comparable cellular density, with no signs of toxicity.

### 3.5 Siponimod Solubility in Porcine Vitreous

To study the possibility of siponimod precipitation in the vitreous, the drug equilibrium solubility in the porcine vitreous was determined and was compared to the equilibrium solubility in PBS (Table 6). Interestingly, at 96 h, the solubility of siponimod in the vitreous (275.93 ± 14.10 μg/mL) was approximately 2.5 times the drug solubility in PBS (99.50 + 4.35 μg/mL). This observation rules out the potential precipitation of siponimod after injection and suggests that the behavior of siponimod at early time points could be attributed to differences in drug diffusion, protein binding, or distribution through the vitreous matrix. Indeed, siponimod is most likely a zwitterion based on the calculated pka values [2.69 (acidic) and 9.15 (basic)], which aids drug dissolution in aqueous medium.

**Table 6:** Siponimod Solubility in Porcine Vitreous and PBS at Different Time Points^a^

time (h)	siponimod concentration (μg/mL)	
	porcine vitreous	PBS
24	340.17 ± 82.08	74.64 ± 10.99
48	310.84 ± 90.32	88.31 ± 5.90
72	275.40 ± 6.46	89.34 ± 6.59
96	275.93 ± 14.10	99.50 ± 4.35

a Values are presented as the average of three independent replicates (*n* = 3) ± standard deviation.

### 3.6 Thermal Stability of Siponimod in Solution and Investigation of Drug Degradation Behavior

**Figure 4:**
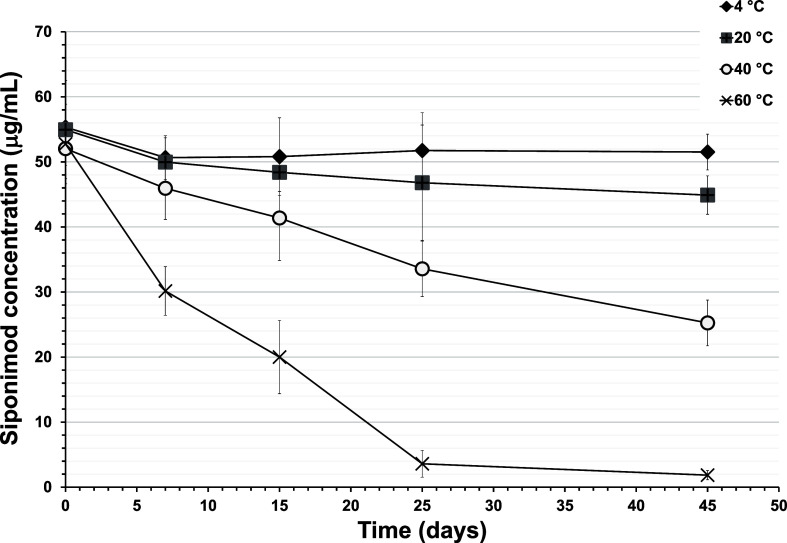
Impact of temperature on the stability of siponimod in solution over time. Graphs show the change in siponimod concentration (in μg/mL) in the solution stored over time (days) at refrigerator temperature (4 °C), room temperature (25 °C), 40, and 60 °C. Three samples were analyzed at each temperature, and the experiment was repeated 3 times (*n* = 3, independent replicates). Data are presented as the mean ± SD.

The concentration of siponimod in PBS (pH 7.2–7.4) was monitored at predetermined time points for up to 45 days at different temperatures to determine the drug’s thermal stability (Figure 4). Siponimod solution incubated at 4 °C did not show a significant loss of the dissolved drug, with no difference between drug concentration on day 45 (51.5 ± 2.8 μg/mL) and day 0 (55.3 ± 0.9 μg/mL). Increasing the temperature led to a significant loss of siponimod, with the amount of degradation dependent on the temperature increase. After 45 days of incubation of siponimod solution at room temperature, 40, and 60 °C, the estimated drug loss was 18.3, 51.5, and 96.1%, respectively (Figure 4). These results indicate the thermal instability of siponimod in solution even when incubated at room temperature. This instability might limit the use of suspension and solution-based dosage forms to deliver the drug for ocular applications^52^ It is important to note that the storage conditions as indicated on the label of the approved solid-dose, tablet formulation (Mayzent), which contains siponimod as a fumarate cocrystal, take the needed precautions to maintain the drug’s stability for the intended storage duration.

Additionally, to identify siponimod’s main degradation product(s), samples stored at 40 °C for 15 (20.5% drug loss) and 45 days (51.5% drug loss) were analyzed using LC–MS. The stressed samples showed a unique compound with weak UV activity that appears at a retention time of 5.00 min with *m*/*z* of 535 (Figures S1–S3). Although this suggests that this compound is a hydration product, considering the increase in molecular weight of 18 over siponimod’s molecular weight, further studies are needed to reveal the compound’s structure. There is no available information about the thermal stability and degradation products of the siponimod form tested here in solution.

## 4 Conclusions

The PK profile of siponimod, a potential novel treatment for ocular neovascular diseases, was characterized in albino rabbits using two different doses of the drug. The PK parameters were estimated using NCA. No considerable difference was observed between the PK profiles of both doses. Considering the low and high doses, the half-lives were 2.80 and 3.88 h, respectively, and neither dose produced any observable signs of retinal toxicity at 24 h and 7 days following injection. This study indicates the lack of short-term toxic effects; however, long-term ocular toxicity studies are warranted. The half-life of siponimod was accurately predicted using models that consider the drug’s molecular weight, lipophilicity, and hydrogen-bond formation ability, suggesting that these factors are important for siponimod clearance. Siponimod stability in solution was compromised by increasing the temperature with the main degradation product identified at a *m*/*z* of 535. The stability data together with siponimod’s half-life in the vitreous underscore the need to develop a sustained drug delivery system that preserves the drug’s integrity if siponimod is to be considered for neovascular ocular disease applications.

## Data Availability

The data sets generated during and/or analyzed during the current study are available from the authors upon reasonable request.

## Supporting Information Available

Mass spectra of siponimod degradation products in solution under different stress conditions (PDF)
