# Supplementary material for: In Vivo Ocular Pharmacokinetics and Toxicity of Siponimod in Albino Rabbits
Source: Mol Pharm. 2024 Jun 10;21(7):3310–20. doi: 10.1021/acs.molpharmaceut.4c00063 (PMC11220753; doi:10.1021/acs.molpharmaceut.4c00063)
Supplement: Supplementary file 1 — mp4c00063_si_001.pdf [file mp4c00063_si_001.pdf]

Supplementary Material

In vivo ocular pharmacokinetics and toxicity of siponimod in albino rabbits

Rasha A. Alshaikh [a], Rania A. Salah El Din [b,c], Rania Gamal Eldin Zaki[d], Christian Waeber [a,e], Katie B. Ryan [a,f]

[a] School of Pharmacy, University College Cork, Cork, Ireland.

[b] Department of Anatomy and Embryology, Faculty of Medicine, Ain Shams University, Cairo, Egypt.

[c] Department of Anatomy and Embryology, Faculty of Medicine, Newgiza University

[d] Department of Ophthalmology, Faculty of Medicine, Ain Shams University, Cairo, Egypt.

[e] Department of Pharmacology and Therapeutics, School of Medicine, University College Cork, Cork, Ireland

[f] SSPC The SFI Research Centre for Pharmaceuticals, School of Pharmacy, University College Cork, Cork, Ireland

Corresponding author

Katie B. Ryan

School of Pharmacy,

University College Cork,

Cork,

Ireland.

Tel 00353-21-4901680

Katie.ryan@ucc.ie

(A)

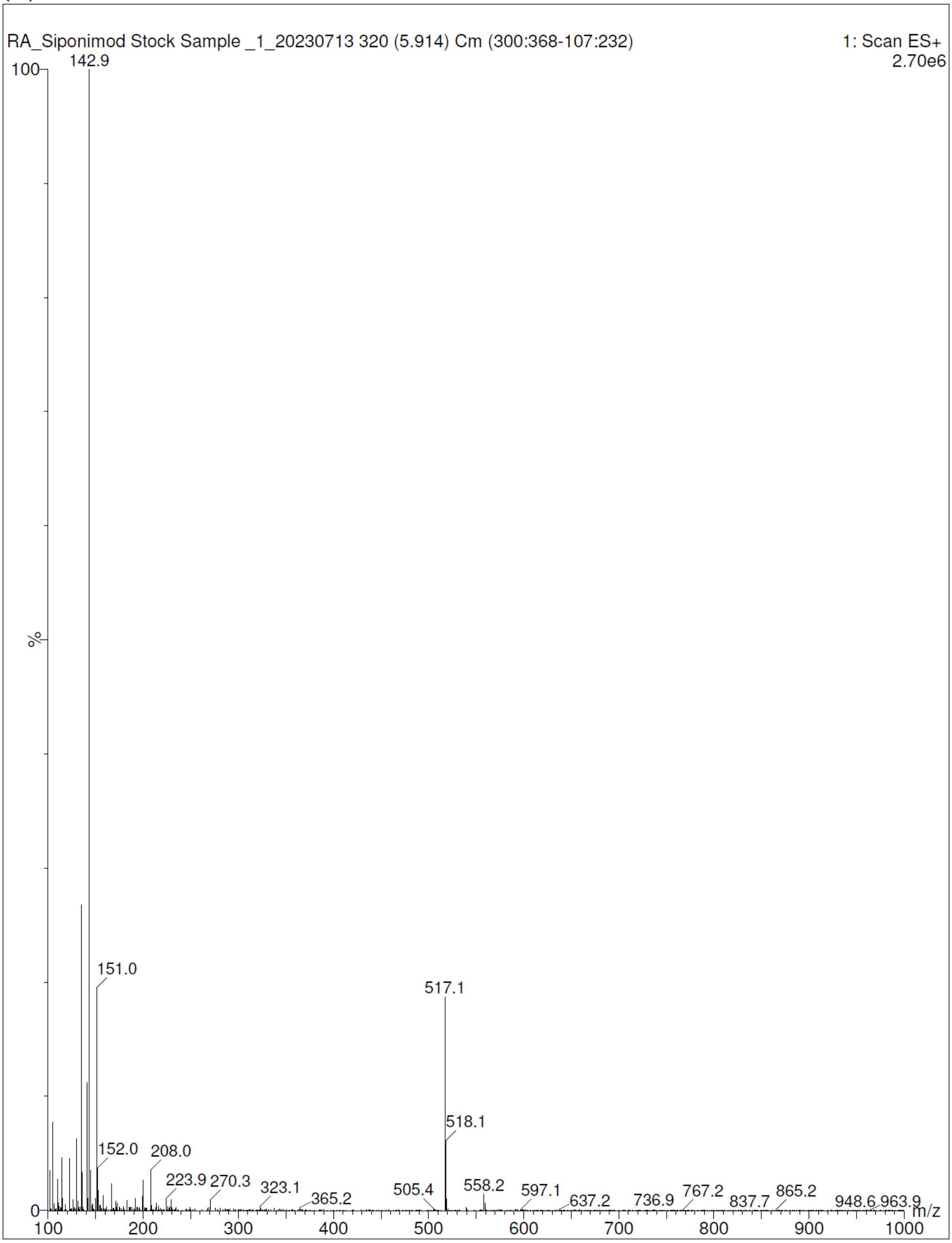
RA_Siponimod Stock Sample _1_20230713 320 (5.914) Cm (300:368-107:232)
1: Scan ES+
2.70e6
100
%
0
142.9
151.0
152.0
208.0
223.9
270.3
323.1
365.2
505.4
517.1
518.1
558.2
597.1
637.2
736.9
767.2
837.7
865.2
948.6
963.9
m/z
100
200
300
400
500
600
700
800
900
1000

(B)

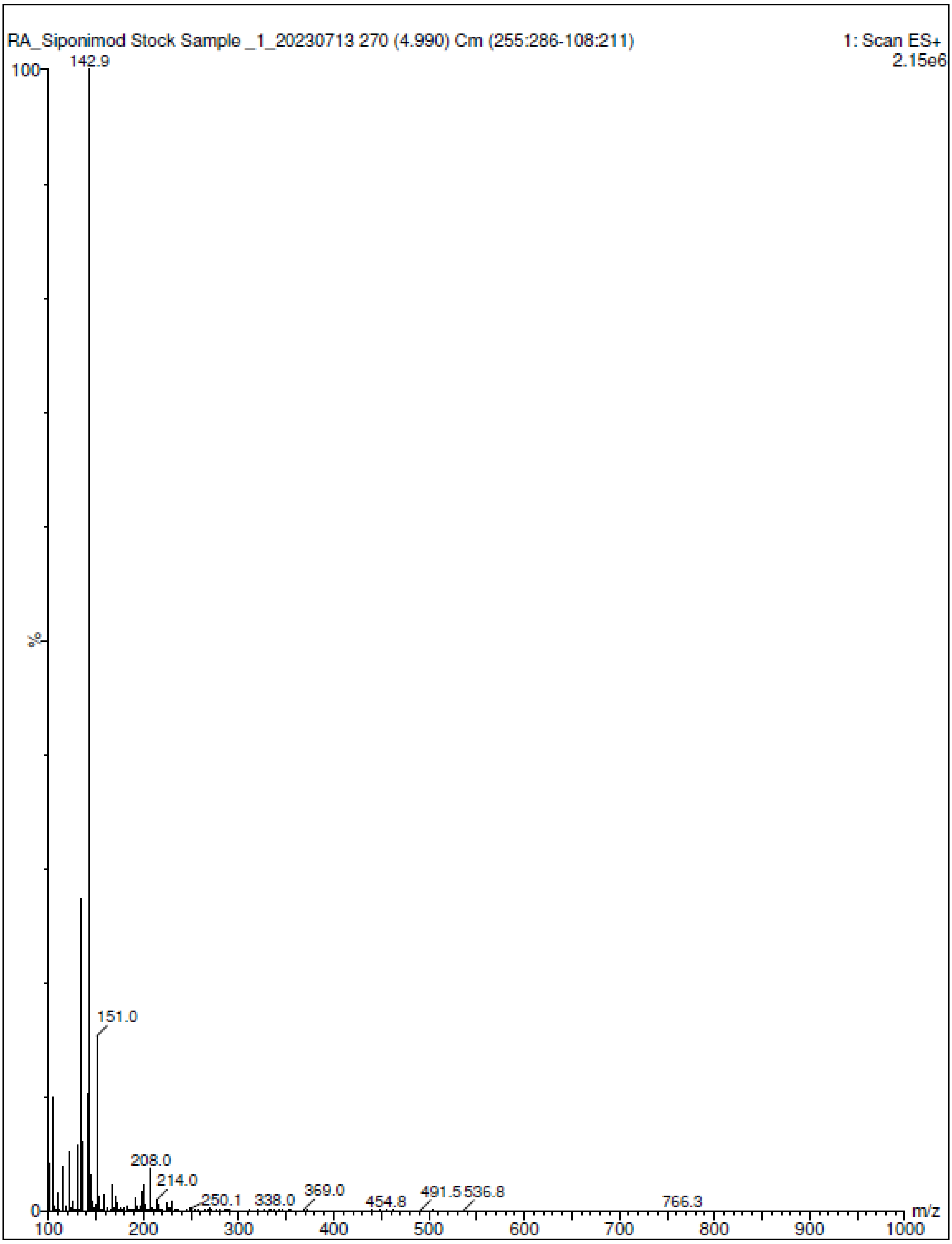

Supplementary Figure 1. Mass spectra of freshly prepared siponimod solution (10 µg/mL). (A) shows siponimod (molecular weight 516.6 g/mol) at m/z 517, with a retention time of 5.79 minutes. (B) shows the mass spectrum at 4.99 minutes with no recognizable peak at m/z of 535.

(A)

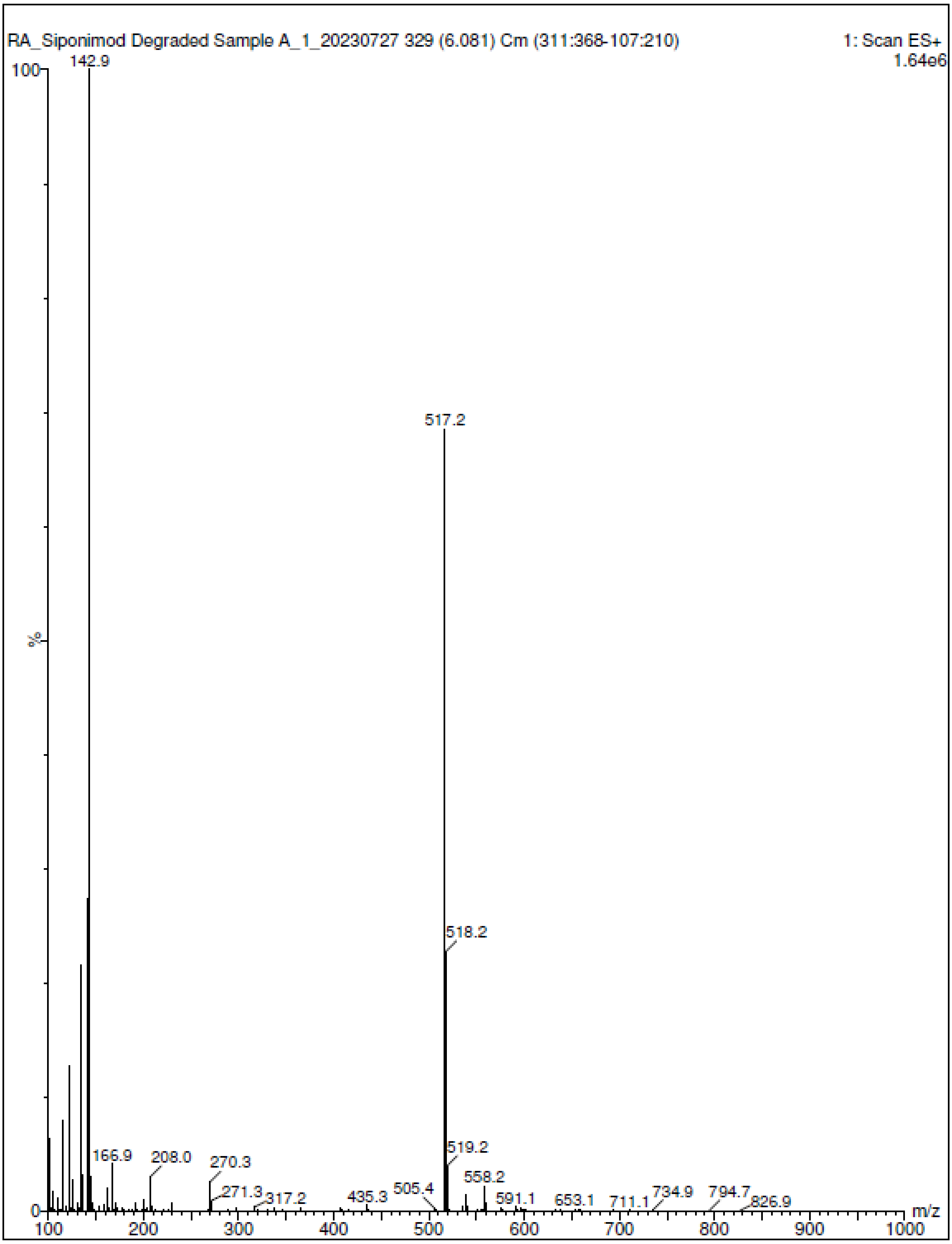

RA_Siponimod Degraded Sample A_1_20230727 329 (6.081) Cm (311:368-107:210)
1: Scan ES+
1.64e6
100
%
0
142.9
517.2
518.2
519.2
558.2
166.9
208.0
270.3
271.3
317.2
435.3
505.4
591.1
653.1
711.1
734.9
794.7
826.9
100
200
300
400
500
600
700
800
900
1000
m/z

(B)

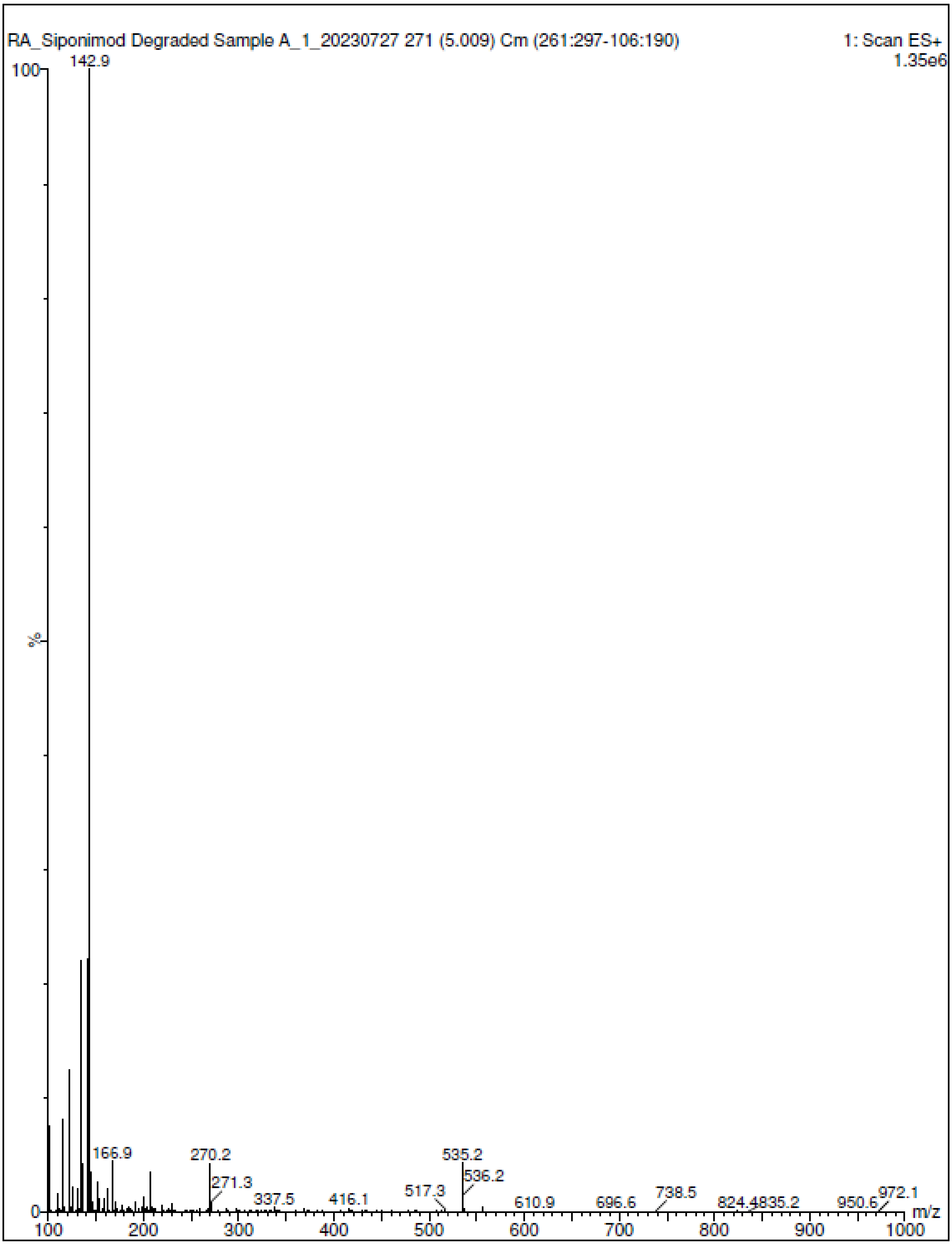

Supplementary Figure 2. Mass spectra of degraded siponimod solution (15 days at 40 °C). (A) mass spectrum showing siponimod at 517 m/z with a retention time of 5.98 minutes. (B) mass spectrum showing the degradation product of siponimod at m/z of 535 with a retention time of 5.00 minutes. This product uniquely appears in the degraded samples.

(A)

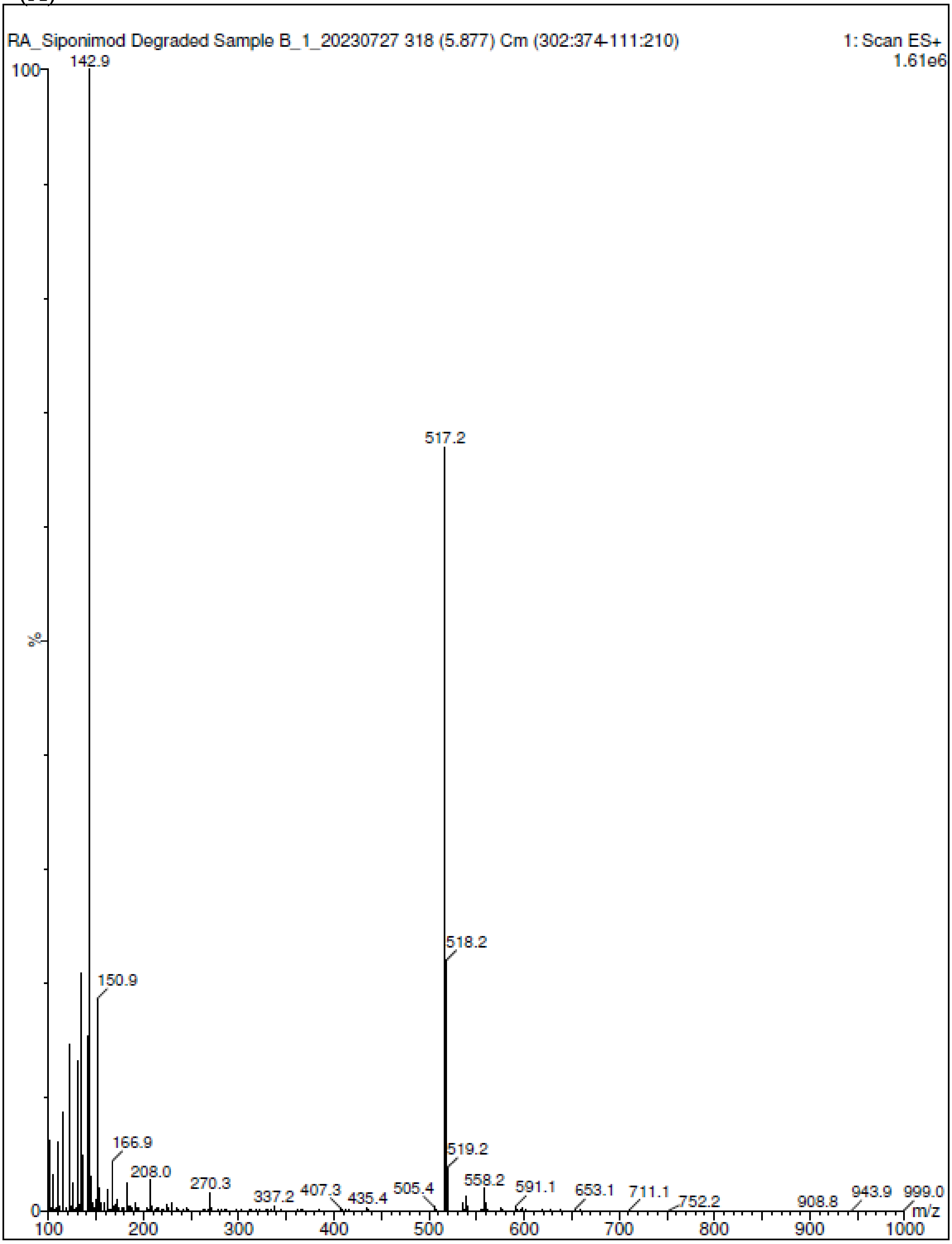
RA_Siponimod Degraded Sample B_1_20230727 318 (5.877) Cm (302:374-111:210)
1: Scan ES+
1.61e6
100
%
0
142.9
517.2
518.2
150.9
166.9
208.0
270.3
337.2
407.3
435.4
505.4
519.2
558.2
591.1
653.1
711.1
752.2
908.8
943.9
999.0
m/z
100
200
300
400
500
600
700
800
900
1000

(B)

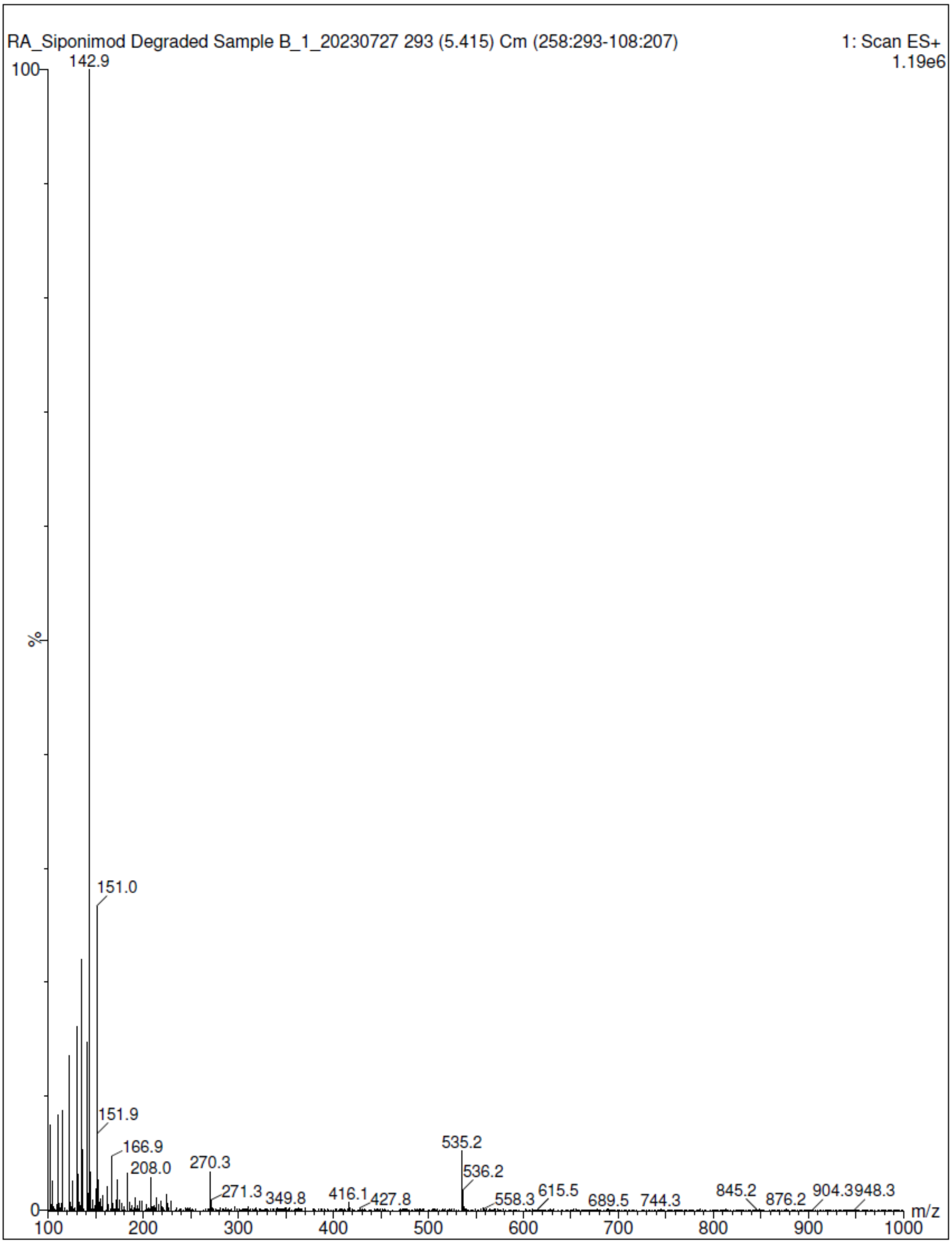

Supplementary Figure 3. Mass spectra of degraded siponimod solution (45 days at 40 ^{o}C). (A) mass spectrum showing siponimod at 517 m/z with a retention time of 5.97 minutes. (B) mass spectrum showing the degradation product of siponimod at m/z of 535 with a retention time of 5.00 minutes. This product uniquely appears in the degraded samples.
